# Thixotropic Supramolecular Pectin-Poly(Ethylene Glycol) Methacrylate (PEGMA) Hydrogels

**DOI:** 10.3390/polym8110404

**Published:** 2016-11-18

**Authors:** Siew Yin Chan, Wee Sim Choo, David James Young, Xian Jun Loh

**Affiliations:** 1 School of Science, Monash University Malaysia, Subang Jaya 47500, Malaysia; siew.chan@monash.edu; 2 Institute of Materials Research and Engineering (IMRE), A*STAR (Agency for Science, Technology and Research), Singapore 138634, Singapore; 3 Faculty of Science, Health, Education and Engineering, University of the Sunshine Coast, Sunshine Coast, QLD 4558, Australia; 4 Department of Materials Science and Engineering, National University of Singapore, Singapore 117576, Singapore; 5 Singapore Eye Research Institute, Singapore 168751, Singapore

**Keywords:** pectin, poly(ethylene glycol) methacrylate, cerium, α-cyclodextrin, supramolecular hydrogel

## Abstract

Pectin is an anionic, water-soluble polymer predominantly consisting of covalently 1,4-linked α-d-galacturonic acid units. This naturally occurring, renewable and biodegradable polymer is underutilized in polymer science due to its insolubility in organic solvents, which renders conventional polymerization methods impractical. To circumvent this problem, cerium-initiated radical polymerization was utilized to graft methoxy-poly(ethylene glycol) methacrylate (mPEGMA) onto pectin in water. The copolymers were characterized by ^1^H nuclear magnetic resonance (NMR), Fourier transform infrared (FTIR) spectroscopy and thermogravimetric analysis (TGA), and used in the formation of supramolecular hydrogels through the addition of α-cyclodextrin (α-CD) to induce crosslinking. These hydrogels possessed thixotropic properties; shear-thinning to liquid upon agitation but settling into gels at rest. In contrast to most of the other hydrogels produced through the use of poly(ethylene glycol) (PEG)-grafted polymers, the pectin-PEGMA/α-CD hydrogels were unaffected by temperature changes.

## 1. Introduction

Pectins are complex carbohydrates derived from dicotyledonous and some monocotyledonous plants [1]. Commercially, pectins are produced from food industry waste [2]. They are mainly extracted with hot dilute mineral acid from citrus peel, apple pomace and to a smaller extent, sugar beet pulp [3,4]. Pectin is a safe food additive with no limit on acceptable daily intake. It is non-toxic, biodegradable and biocompatible [5,6]. Its applications are diverse spanning the food, pharmaceutical, cosmetic and polymer industries [7,8,9]. Pectins are primarily utilized as emulsifiers, gelling agents, glazing agents, stabilizers or thickeners [10]. 

This family of polysaccharides consists of galacturonic acid (GalA) units covalently-linked by α-(1→4) glycosidic bonds and are classified according to the degree of methylation (DM). DM is defined as the molar ratio of methyl-esters present relative to the total moles of GalA units. It is the major parameter affecting gelling [11]. Pectins are classified as either high methoxy pectin (HMP) with a DM > 50% or low methoxy pectin (LMP) with a DM < 50%. HMP gels in high co-solute concentration with acid through a combination of hydrophobic forces and hydrogen bonding [12]. LMP gels in the presence of divalent metal cations such as calcium over a broad range of pH and the gels are formed through ionic cross-linking between free carboxylate groups in an arrangement known as the egg-box model [13]. 

We have an interest in grafting pectin with synthetic polymers to improve its processability in organic solvents and to impart biocompatibility and biodegradability to the resulting copolymer for use in biomedical applications. Herein, we describe pectin-poly(ethylene glycol) methacrylate (PEGMA) copolymer and subsequently generated supramolecular hydrogels by threading the poly(ethylene glycol) (PEG) tendrils with α-cyclodextrin (α-CD) units. Harada et al. [14] first reported supramolecular hydrogel formed by threading α-cyclodextrin toroids onto PEG of high molecular weight. This PEG-graft forms physical cross-linking points via the aggregation of the inclusion complexes, yielding supramolecular hydrogels [15,16,17,18,19]. 

Pectin is a hydrophilic polymer and insoluble in all organic solvents. Most polymerization methods require organic solvents, making it a challenge to graft pectin with synthetic polymers. We have for the first time utilized a redox polymerization reaction in water to graft PEGMA onto pectin and have thereby generated pectin supramolecular gels with unique rheology.

## 2. Materials and Methods

Ammonium cerium (IV) sulfate dihydrate, apple pectin and sodium nitrate were purchased from Sigma-Aldrich, Singapore, Singapore. Methoxy-poly(ethylene glycol) methacrylate (mPEGMA) with Mn of 10,000 g·mol^−1^ was purchased from Sinopeg, Xiamen, China. α-Cyclodextrin (α-CD) was purchased from TCI, Kawaguchi, Japan. All reagent and solvents were used as received.

### 2.1. Synthesis of Pectin-PEGMA Copolymer, P-10K

**Figure 1:**
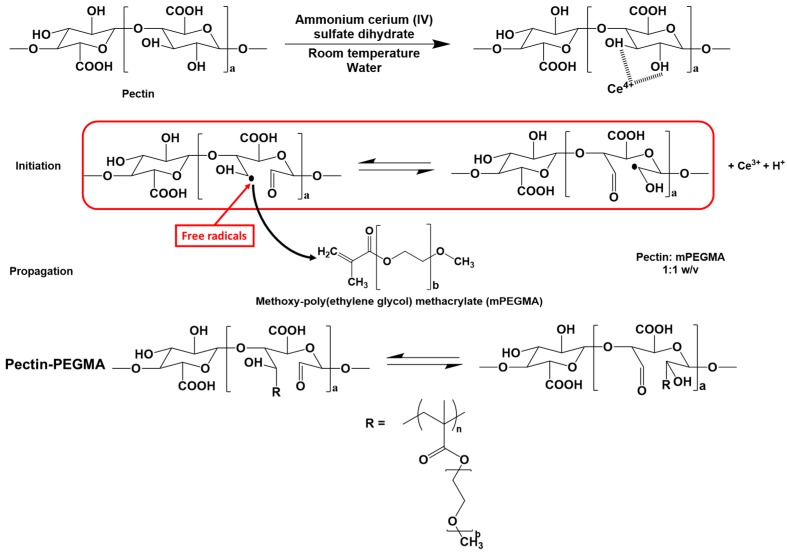
Mechanism of cerium-initiated radical polymerization.

Ammonium cerium (IV) sulfate dihydrate (0.005 M, based on the final volume of solution) was added into a pectin solution (1% *w*/*v* in water). The solution was stirred at room temperature for 2 h. mPEGMA (1% *w*/*v*) was then added. The reaction mixture was stirred at room temperature for 48 h. Chloroform was added and the resulting mixture was left to settle. Pectin-PEGMA was obtained in 61.5% yield by precipitating the white emulsion layer in hexane followed by drying under vacuum at 40 °C. The mechanism of cerium-initiated radical polymerization is shown in Figure 1.

### 2.2. Polymer Characterisation

^1^H Nuclear magnetic resonance (NMR) spectra were recorded on a JEOL 500 MHz NMR spectrometer (JEOL, Tokyo, Japan) at room temperature. The ^1^H NMR measurements were carried out with an acquisition time of 4.37 s, a pulse repetition time of 9.37 s and a 90° pulse width. Measurements were done with 16 scans and chemical shifts were referred to the solvent peak (δ = 4.79 ppm for deuterium oxide, D_2_O).

Fourier transform infrared (FTIR) spectra of the pellet samples were recorded on a Perkin Elmer Spectrum 2000 FTIR spectrometer (Perkin Elmer, Waltham, MA, USA); 64 scans were signal-averaged with a resolution of 4 cm^−1^ at room temperature. Pellets were prepared by coating the samples with potassium bromide.

### 2.3. Thermal Analysis

Thermogravimetric analysis (TGA) was performed on a TA Instruments TGA Q500 (TA Instruments, New Castle, DE, USA). Samples were heated from room temperature to 900 °C at a rate of 20 °C·min^−1^ under continuous nitrogen purge with a flow rate of 40 mL·min^−1^.

### 2.4. Preparation of Pectin-PEGMA/α-CD Hydrogels

**Figure 2:**
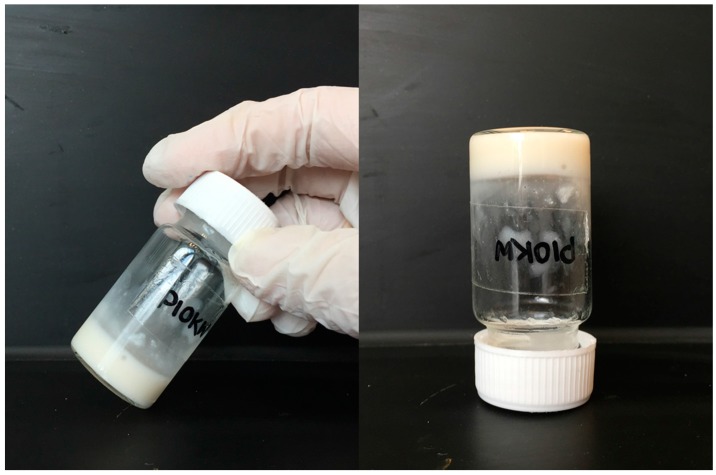
The preparation of pectin-PEGMA/α-CD gels.

Solutions of α-CD in water were added into pectin-PEGMA solutions of different compositions. The resultant mixtures were sonicated and left to stand at room temperature. Gel formation was observed at 24 h (Figure 2). The sol-gel transition for P-10K was determined by plotting α-CD concentration versus polymer concentration over the polymer to α-CD solution composition range of 1%–10% *w*/*v*, permitting determination of the critical gelation concentration.

### 2.5. Rheology Studies

The rheological behavior of pectin-PEGMA/α-CD gels was investigated using a Discovery DHR-3 hybrid rheometer (TA Instruments, New Castle, DE, USA) with flat plate geometries (diameters of 20 and 40 mm) in steady and dynamic modes. All the tests were performed using a flat plate geometry with diameter of 40 mm unless otherwise stated. Amplitude sweeps were performed under oscillatory shear at strain of 0.01%–100% to ensure that subsequent data were collected in the linear viscoelastic region (LVR). Frequency sweeps were then performed in the range of 0.01–50 Hz under oscillatory shear at a strain of 0.05%. Reversibility of the hydrogels was determined by amplitude sweeps at 2 points (0.05% and 25% strain), 5 and 2.5 min, respectively, at each strain point, for 3 cycles. All tests were performed at 25 °C (room temperature) and 37 °C to mimic body temperature. Temperature sweeps were performed using a flat plate geometry with diameter of 20 mm at a strain of 0.5% and frequency of 1 Hz in the temperature range of 10–40 °C, with a ramp rate of 5 °C·min^−1^.

## 3. Results

### 3.1. Synthesis and Characterization of Pectin-PEGMA Copolymer, P-10K 

Pectin-PEGMA copolymer (P-10K) was prepared by radical polymerization in distilled water using ammonium cerium (IV) sulfate dihydrate as a redox initiator. Chloroform was added to extract excess PEGMA. Three layers formed in settling; a cloudy brown solution, a white middle layer emulsion and at the bottom a transparent chloroform containing the unreacted mPEGMA.

**Figure 3:**
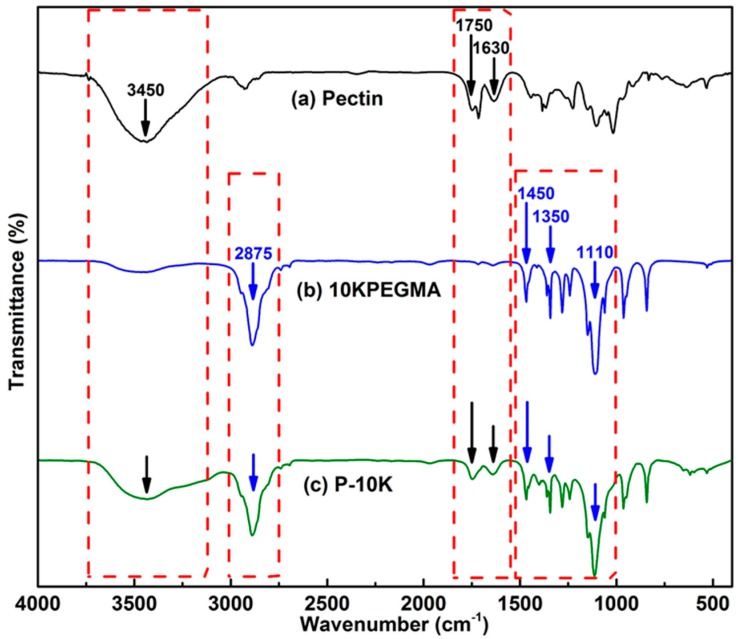
FTIR spectra of the precursors (pectin and 10KPEGMA) and of copolymer P-10K.

^1^H NMR spectroscopy in D_2_O was performed on the middle emulsion layer, but proved inconclusive (Figure S1). FTIR, however, contained absorption characteristics of pectin and mPEGMA (10KPEGMA). Pectin exhibits a broad peak at around 3450 cm^−1^, arising from hydroxyl group stretching (O–H) (Figure 3) [20]. Absorptions at 1750 and 1630 cm^−1^ were assigned to the carboxylic acid and ester groups (C=O stretches) of the pectin polymers [21]. 10KPEGMA displays a broad peak around 3450 cm^−1^ but not as intensive as for pectin. The FTIR spectrum of 10KPEGMA also contained distinctive C–H stretches at 2875 cm^−1^. CH_2_ (bending) stretches at 1450 cm^−1^, CH_3_ (bending) stretches at 1350 cm^−1^ and C–O stretches at 1110 cm^−1^ were also observed. The FTIR spectrum obtained for 10KPEGMA matched those reported by Wang et al. [22] and Bagheri et al. [23].

### 3.2. Thermal Analysis

**Figure 4:**
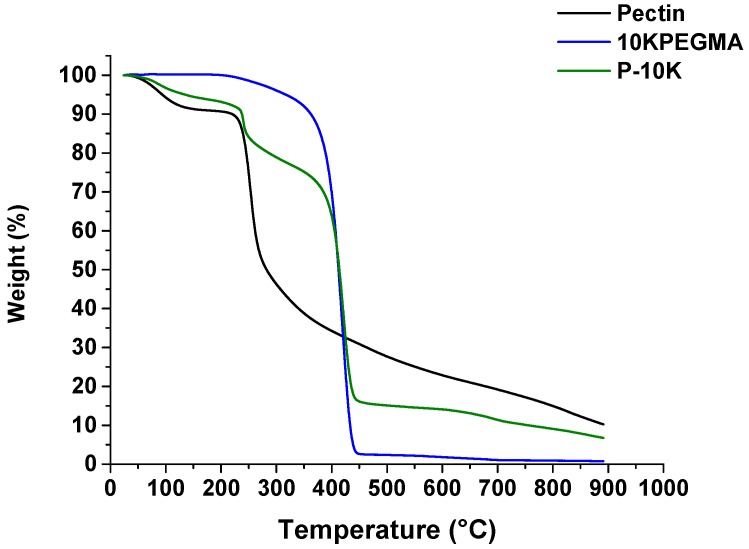
TGA curves of the precursors (pectin and 10KPEGMA) and copolymer P-10K.

Thermal stabilities of the precursors and copolymer were studied by TGA. The degradation temperatures were determined from the peak of derivative weight curves and are summarized in Table 1. There were two degradation temperatures recorded for pectin; 86.33 and 253.33 °C (Figure 4). The first degradation temperature corresponds to the loss of water. 10KPEGMA exhibited a degradation temperature of 413.53 °C. TGA of P-10K revealed three degradation temperatures, corresponding to those for pectin and mPEGMA. The amount of pectin in P-10K was calculated to be around 17% (Table 1). 

### 3.3. Critical Gelation Concentration Determination 

**Figure 5:**
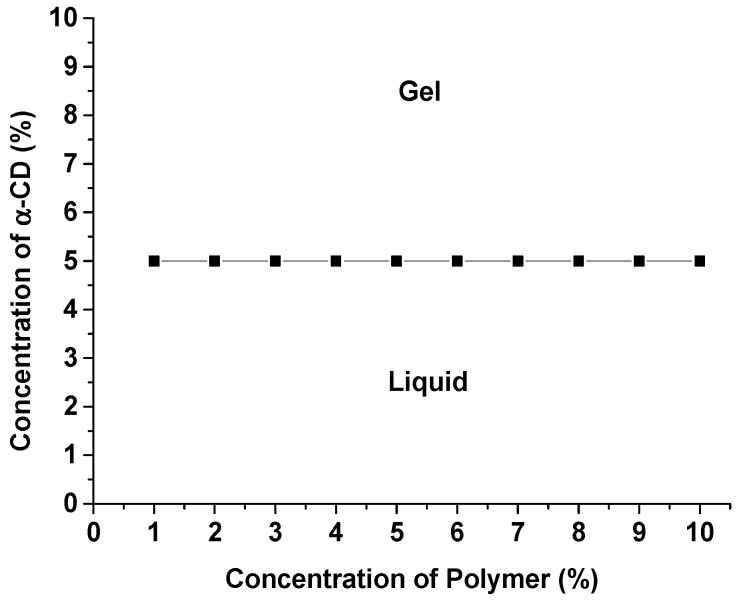
Sol-gel transition graph for different compositions of P-10K/α-CD.

Aqueous solutions of α-CD were added to P-10K solutions and the mixtures became gradually opaque. The mixtures turned white and formed gels after a time period. The effects of the polymer and α-CD concentrations on gelling behavior were studied by mixing a range of polymer and α-CD compositions (1%–10% *w*/*v*). Hydrogel could be formed at a low polymer concentration of 1% (*w*/*v*) (Figure 5). The copolymer gelled at an optimum ratio of 1% (*w*/*v*) polymer and 5% (*w*/*v*) α-CD. Ye et al. have reported that higher cyclodextrin concentrations increase the gel strength [24].

### 3.4. Rheology of Pectin-PEGMA/α-CD Hydrogel

**Figure 6:**
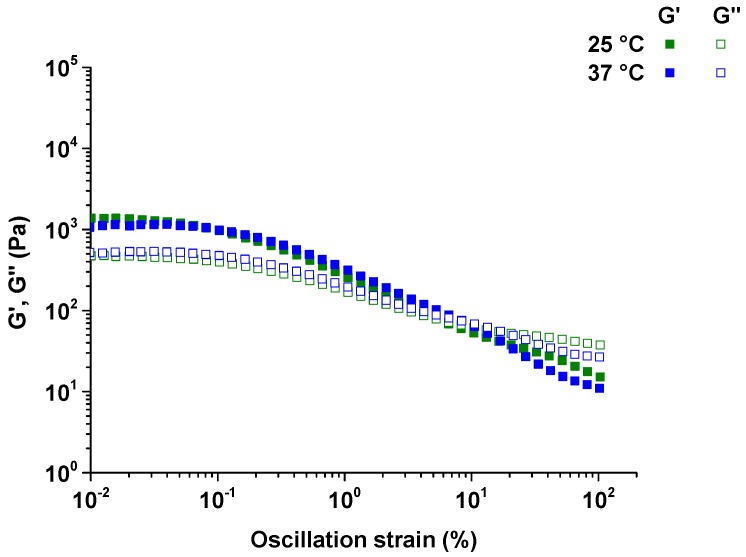
Amplitude sweeps performed from 0.01% to 100% of oscillation strain at 25 and 37 °C on hydrogels prepared with 10% P-10K and 10% α-CD.

The rheology of hydrogels prepared with 10% P-10K and 10% α-CD was investigated. Amplitude sweeps to measure shear strain were in the range of 0.01%–100% at temperatures of 25 °C (room temperature) and 37 °C (body temperature). The hydrogels proved to be highly structured, true gels with storage moduli (*G*’) greater then loss moduli (*G*”) at low shear strain (Figure 6). As the shear strain increased, *G*’ began to decrease at a rate faster than that of *G*”. As the oscillation strain increased from 0.01% to 100%, the hydrogels changed from a gel (*G*’ > *G*”) to a liquid (*G*’ < *G*”). The critical strain of the hydrogel was around 7%–11% for P-10K. At a shear strain greater than the critical strain, *G*’ dropped below *G*”, indicating that the gel network structures had been disrupted by shearing. The linear viscoelastic region (LVR) of the hydrogels was 0.01% to 1% strain [25].

**Figure 7:**
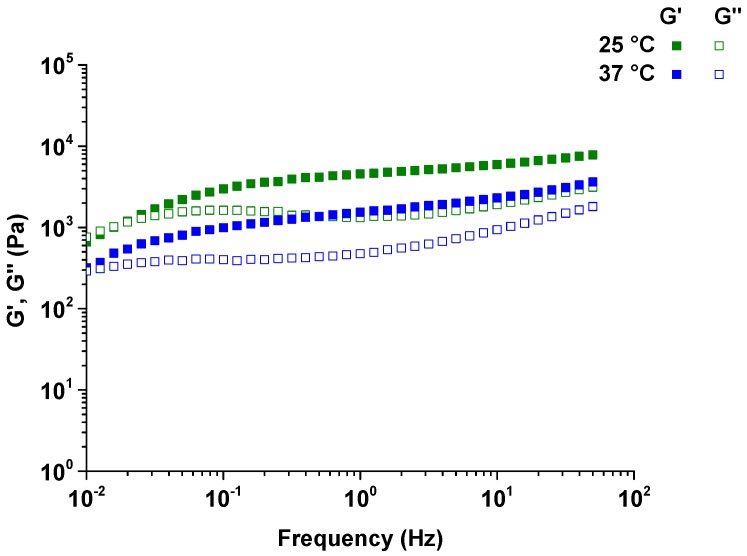
Frequency sweeps performed from 0.01 to 50 Hz of oscillation strain at 25 and 37 °C on hydrogels prepared with 10% P-10K and 10% α-CD.

Frequency sweeps were performed on the hydrogels from 0.01–50 Hz at 0.05% strain at 25 and 37 °C. *G*’ were higher than *G*” and both the moduli were dependent on frequency at both 25 and 37 °C (Figure 7). Frequency sweeps provide information on the effect of colloidal forces or the interaction among particles [26]. As frequency increased, there appeared to be no interaction between particles, presumably because of sufficient separation to eliminate interactions and/or collisions. 

### 3.5. Thixotropic Properties of Pectin-PEGMA/α-CD Hydrogels

**Figure 8:**
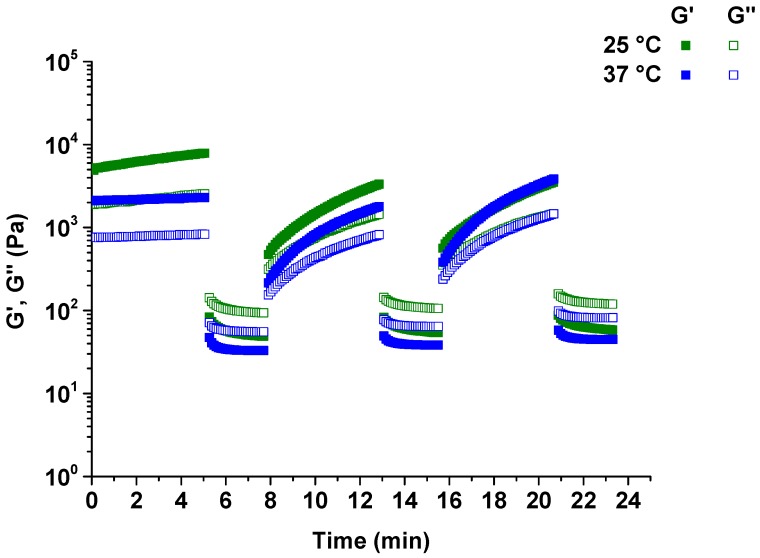
Amplitude sweeps at 0.05% and 25% strains performed instantaneously for three cycles at 25 and 37 °C on hydrogels prepared with 10% P-10K and 10% α-CD.

Instantaneously varying strain at 0.05% and 25% in amplitude sweeps for three cycles indicated that P-10K/α-CD hydrogels recovered their original gel structure (Figure 8) [27]. *G*’ was higher than *G*” during the first 5 min of 0.05% strain and the gel was immediately sheared into a liquid where *G*’ < *G*” during the 2.5 min of 25% strain. The liquid then reverted back to gel (*G*’ > *G*”) when the strain was reduced to 0.05%. However, the hydrogel required some recovery time to revert back to the original gel form. The internal network structure could be broken down by shearing, and required time to rebuild, as shown by the coincides of *G*’ and *G*” at each 0.05% strain point after being sheared at 25% strain. This shear–recovery phenomenon was repeatable for all the hydrogels at a minimum of three cycles, proving the thixotropy of P-10K/α-CD hydrogels.

### 3.6. The Effect of Temperature on Pectin-PEGMA/α-CD Hydrogels

**Figure 9:**
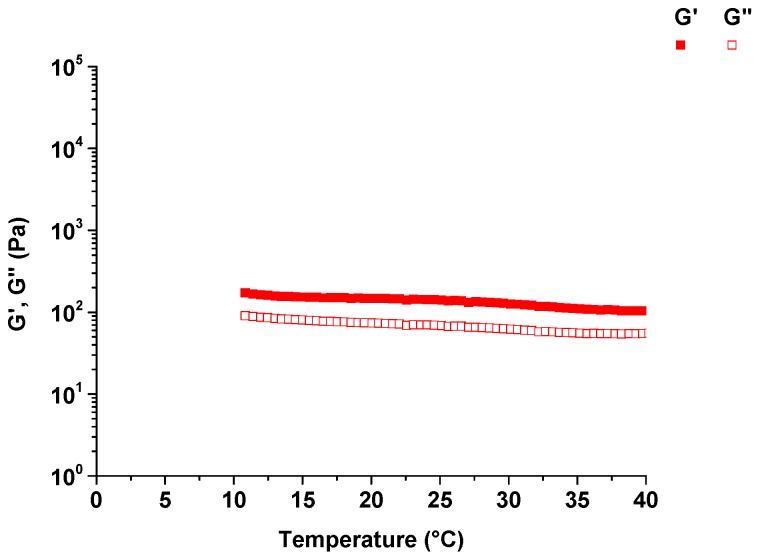
Temperature sweep performed from 10 to 40 °C on hydrogel prepared with 10% P-10K and 10% α-CD.

Temperature sweeps were performed to probe the temperature responsiveness of P-10K/α-CD hydrogels (Figure 9) and demonstrated that these gels were not affected by temperature from 10 to 40 °C. *G*’ was higher than *G*” and the moduli remained almost constant over this range. 

## 4. Discussion

Pectin-PEGMA copolymer was successfully synthesized through cerium-initiated radical polymerization, as evidenced by FTIR spectroscopy (Figure 3) and TGA (Figure 4). The latter, in particular, was diagnostic, indicating the presence of two components in P-10K and a shift in the degradation temperature of the copolymer relative to its component precursors. Based on the weight change determined from these TGA curves (Table 1), it can be concluded there was 17% pectin in P-10K (Table 1). The FTIR spectra of this block copolymer supported the presence of pectin as a minority component (Figure 3).

**Figure 10:**
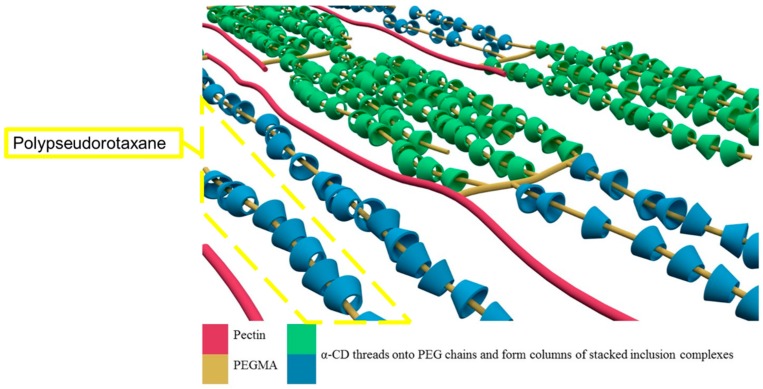
Threading of α-CD onto PEG chains to form columns of stacked inclusion complexes.

**Figure 11:**
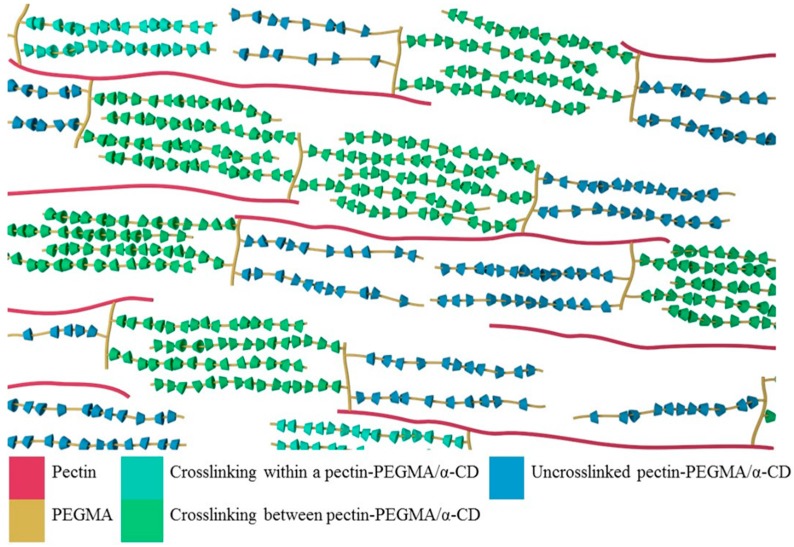
Polypseudorotaxanes inter- or intra-crosslink with each other via hydrogen bonding.

**Figure 12:**
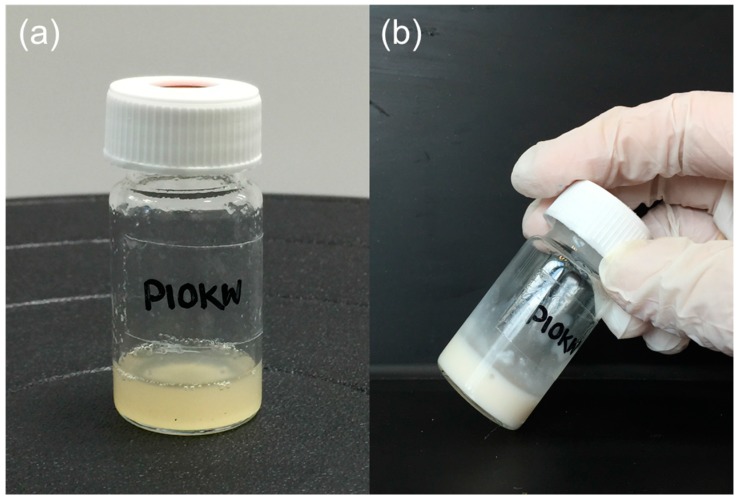
Pectin-PEGMA solution turned white after the addition of α-CD: (**a**) original 10% P-10K solution; and (**b**) 10% P-10K solution after the addition of α-CD (10%).

Pectin-PEGMA copolymer was able to form gels with α-CD (Figure 5). The α-CD threaded onto PEG tendrils to form stacked inclusion complexes (Figure 10) [28,29] that interact with each other to aggregate via hydrogen bonding [30]. These polypseudorotaxanes can inter- or intra-crosslink with each other while some chains of stacked inclusion complexes may remain separated (Figure 11). A control experiment involving mPEGMA alone turned opaque and eventually white when α-CD was added, but no gel was formed (Table 2). Inclusion complexes still formed between α-CD and PEG chains. However, the columns of threaded PEG chains remained free with no gelation [31]. P-10K required 5% α-CD to gel (Figure 5). Upon addition of 10% α-CD into 10% 10KPEGMA and 10% P-10K solutions, respectively, the co-polymer sample gelled, but not mPEGMA (Figure 12).

**Figure 13:**
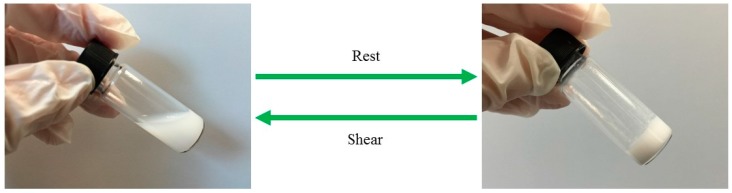
Thixotropic behavior of pectin-PEGMA/α-CD hydrogels.

The response of pectin-PEGMA/α-CD hydrogel to shear strain and its dependence on frequency demonstrated shear-thinning. The higher the strain percentage was, the more liquid-like the hydrogel became. The time oscillation test further demonstrated that the hydrogel was thixotropic. Thixotropic materials provide a gel consistency when at rest and flow when shear is introduced (Figure 13). Pectin-PEGMA/α-CD hydrogel was sheared beyond critical strain, but reverted back to gel on resting. Most PEG-grafted polymers are temperature-sensitive, with the network structure disrupted by an increase in temperature [15,19]. High temperature may disrupt the non-covalent interactions in supramolecular hydrogels, causing α-CD to de-thread from PEG chains [15]. The physical cross-linked network will then collapse. This effect was not observed for pectin-PEGMA/α-CD hydrogel which maintained its gel consistency from 10 to 40 °C. We hypothesize that pectin may confer thermal stability to the pectin-PEGMA/α-CD hydrogels. In support of this proposition, it has been reported that heat treatment of milk does not affect the ability of pectin to stabilize particles such as casein and denatured whey complex [32]. 

In conclusion, pectin-PEGMA (P-10K) copolymer was successfully synthesized using cerium-initiated radical polymerization in water. This process is a safer, greener, eco-friendlier way to graft polymers. α-CD was incorporated into pectin-PEGMA copolymer and supramolecular gels were formed. The copolymer gelled at low polymer concentration (1%) with 5% of α-CD. The total concentration of polymer could be lowered to achieve a desired consistency of gel. These supramolecular hydrogels were thixotropic, i.e., the gel could be sheared to liquid and reverted back to gel at rest. Industrially, a thixotropic material is easier to process or transfer without disrupting the consistency. Interestingly, pectin-PEGMA/α-CD hydrogels were not affected by temperature from 10 to 40 °C, unlike the hydrogels of other PEG-grafted polymers [15,19,33,34]. This supramolecular hydrogel has potential to be developed for cosmetic, body and hair care products, or even as an injectable pharmaceutical incipient [35,36]. We are currently evaluating the biocompatibility and biodegradability of pectin-PEGMA and exploring the response of this material to other external stimuli.

## Figures and Tables

**Table 1:** Decomposition temperatures (*T*_d_) of the precursors (pectin and 10KPEGMA) and copolymer P-10K.

Sample	*T*_d_ (°C)	Weight change (%)
Pectin	86.33	8.95
	253.33	80.82
10KPEGMA	418.07	99.02
P-10K	85.89	5.74
	240.29	16.70
	421.19	62.91

**Table 2:** Ten percent 10KPEGMA and 10% P-10K solutions with 10% α-CD.

Sample	Gel
10KPEGMA	✗
P-10K	✓

## Supplementary Materials

The following are available online at www.mdpi.com/2073-4360/8/11/404/s1. Figure S1: 500 MHz ^1^H NMR spectra of the precursors (pectin and 10KPEGMA) and of copolymer P-10K.
