# Supplementary material for: Thixotropic Supramolecular Pectin-Poly(Ethylene Glycol) Methacrylate (PEGMA) Hydrogels
Source: Polymers (Basel). 2016 Nov 18;8(11):404. doi: 10.3390/polym8110404 (PMC6432130; doi:10.3390/polym8110404)
Supplement: Supplementary file 1 [file polymers-08-00404-s001.pdf]

Supplementary Materials: Thixotropic Supramolecular Pectin-Poly(Ethylene Glycol) Methacrylate (PEGMA) Hydrogels

Siew Yin Chan, Wee Sim Choo, David James Young and Xian Jun Loh

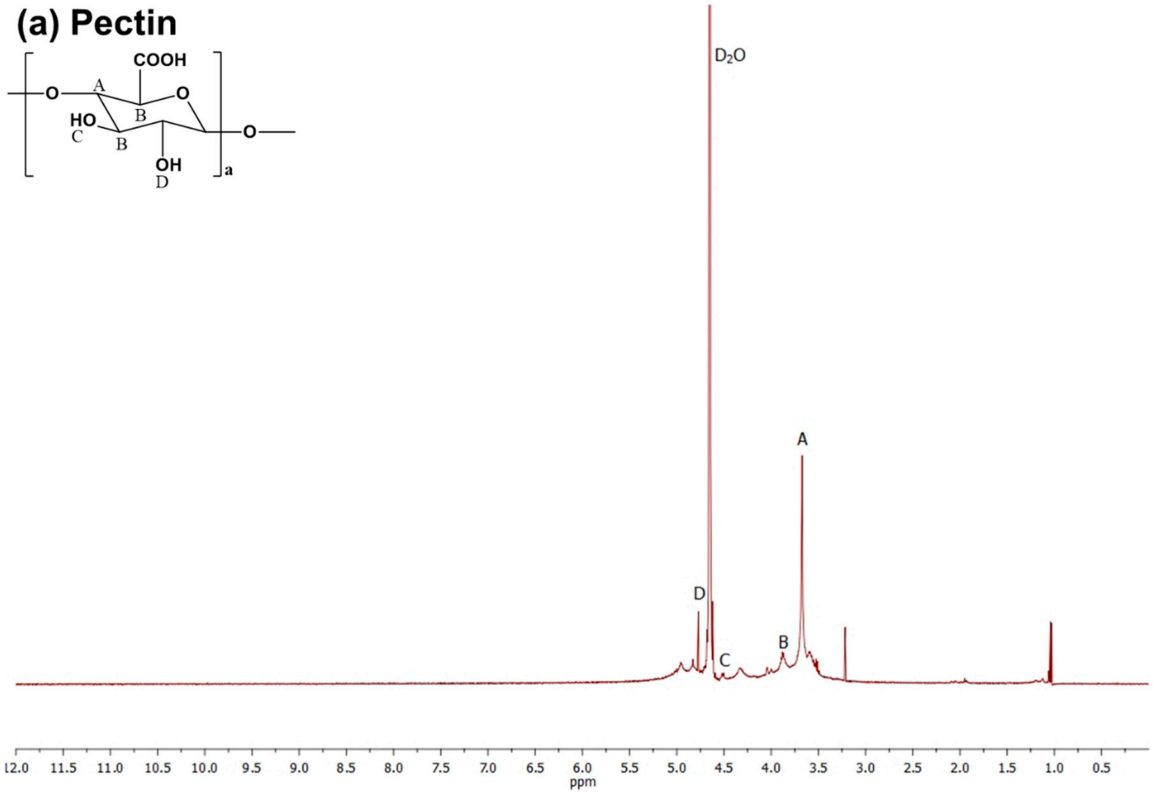

Figure S1. *Cont.*

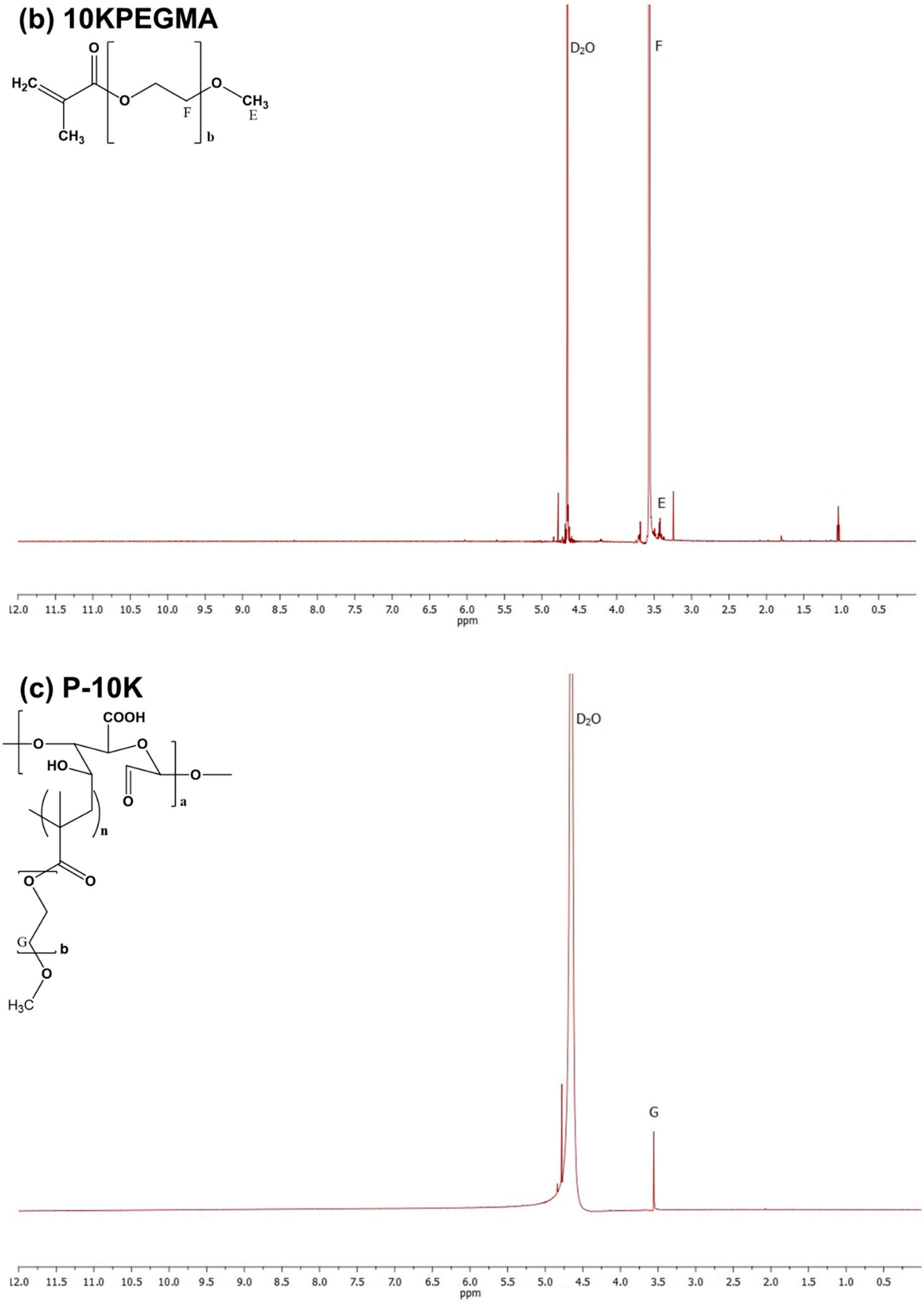

Figure S1. 500 ^{1}H NMR spectra of the precursors (pectin and 10KPEGMA) and copolymer P-10K.
